# Optimal Sequence of Irinotecan and Oxaliplatin-Based Regimens in Metastatic Colorectal Cancer: A Population-Based Observational Study

**DOI:** 10.1371/journal.pone.0135673

**Published:** 2015-08-14

**Authors:** Chieh-Lin Jerry Teng, Chen-Yu Wang, Yi-Huei Chen, Ching-Heng Lin, Wen-Li Hwang

**Affiliations:** 1 Division of Hematology/Medical Oncology, Department of Medicine, Taichung Veterans General Hospital, Taichung, Taiwan; 2 Department of Medicine, Chung Shan Medical University, Taichung, Taiwan; 3 Department of Life Science, Tunghai University, Taichung, Taiwan; 4 Department of Intensive Care, Taichung Veterans General Hospital, Taichung, Taiwan; 5 Department of Education and Research, Taichung Veterans General Hospital, Taichung, Taiwan; University Campus Bio-Medico, ITALY

## Abstract

The optimal sequence of irinotecan and oxaliplatin-based regimens for metastatic colorectal cancer remains unclear. We conducted a population-based observational study by retrospectively reviewing records from Taiwan’s National Health Insurance Research Database to explore this issue. Patients aged ≥20 years with metastatic colorectal cancer newly diagnosed between 2004 and 2008 (n = 9490) were enrolled in current study. Among these 9490 patients, 3895 patients (41.04%) did not receive any chemotherapy within the first three months after catastrophic illness registration. Patients who received best supportive care were older and had higher Charlson comorbidity indexes and incidences of comorbidities than those who received irinotecan-based regimens, oxaliplatin-based regimens, and 5-fluorouracil/capecitabine alone. Patients who received irinotecan followed by oxaliplatin-based regimens and those who received the reverse sequence were further stratified into arm A (n = 542) and arm B (n = 1156), respectively. The median first time to next treatment was not significantly different between arm A and arm B (210 days vs. 196 days; p = 0.17). However, the median second time to next treatment was longer in arm A than in arm B (155 days vs. 123 days; p = 0.006), which translated into a better overall survival (487 days vs. 454 days; p = 0.02). The crossover rate was higher in arm A than in arm B (47.84% vs. 41.61%; p<0.001). Multivariate Cox regression analyses showed that overall survival was comparable between the two chemotherapy sequences (p = 0.27). Our study suggested that irinotecan followed by oxaliplatin-based regimens might be a better chemotherapy treatment option for metastatic colorectal cancer than the reverse sequence given the higher crossover rate and potential overall survival benefit.

## Introduction

Colorectal cancer is the fourth most common cancer in the United States, with more than 140,000 new cases diagnosed each year [1]. Approximately half of the patients with colorectal cancer will eventually develop inoperable metastatic disease and require palliative chemotherapy. Higher response rate and prolongation of progression-free survival (PFS) and overall survival (OS) are the treatment goals of palliative chemotherapy for inoperable metastatic colorectal cancer (mCRC). Irinotecan or oxaliplatin combined with fluorouracil/leucovorin is currently considered to be the chemotherapy backbone for mCRC [2]. Oxaliplatin plus 5-fluorouracil/leucovorin demonstrated a superior PFS compared with 5-fluorouracil/leucovorin alone in mCRC patients [3]. Moreover, mCRC patients receiving irinotecan plus 5-fluorouracil/leucovorin have been shown to have a longer PFS and OS than those receiving 5-fluorouracil/leucovorin alone [4].

Unfortunately, disease progression is almost unavoidable after front-line chemotherapy. Second-line oxaliplatin and irinotecan-based regimens may be reasonable options for mCRC patients who have failed front-line irinotecan and oxaliplatin-based regimens, respectively. The optimal sequence of irinotecan and oxaliplatin-based regimens for mCRC remains a matter of debate. In a phase III randomized Groupe Coopérateur Multidisciplinaire en Oncologie (GERCOR) study, PFS and OS were similar between patients treated with FOLFIRI (leucovorin, 5-fluorouracil, and irinotecan) followed by FOLFOX6 (leucovorin, 5-fluorouracil, and oxaliplatin) and those treated with the reverse sequence[5], suggesting that the sequence of oxaliplatin and irinotecan-based regimens does not significantly impact patient outcome. Notably, the crossover rate after disease progression was higher for patients treated with first-line FOLFIRI than for patients treated with first-line FOLFOX6.

In the past decade, various biological therapies for mCRC have emerged and have been integrated into cytotoxic regimens. Addition of the anti-vascular endothelial growth factor monoclonal antibody bevacizumab to irinotecan-based chemotherapeutic regimens has been shown to improve both PFS and OS in mCRC patients [6]. FOLFIRI in combination with the anti-epidermal growth factor receptor monoclonal antibody cetuximab has been suggested to improve PFS compared with FOLFIRI alone in *KRAS* wild-type mCRC patients [7]. Because much of the focus of mCRC treatment has shifted toward the combined use of biological and cytotoxic therapies in upfront settings, optimal chemotherapeutic strategies for mCRC have become an unmet clinical need and require further investigation.

The aim of this study was to investigate the real-world chemotherapeutic strategies for mCRC before the era of biological therapy using nationwide population-based data. In addition, patient outcomes were compared between different sequences of irinotecan and oxaliplatin-based regimens.

## Materials and Methods

### Data source

The population for this study was derived from the Longitudinal Health Insurance Database, which is based on the Taiwanese National Health Insurance Research Database. This database contains comprehensive clinical records for insured persons. Patient data obtained from the clinical records included anonymized identification numbers, demographic characteristics, inpatient and outpatient dates, diagnostic codes (International Classification of Disease, 9th Revision, Clinical Modification [ICD-9-CM]), and prescriptions ordered between March 1995 and December 2010. More than 99% of the entire population of Taiwan is included in this database. The details of this population-based database have been described previously [8]. The study protocol was approved by the institutional review board of Taichung Veterans General Hospital, and the requirement for written informed consent from the participants was waived by the institutional review board of Taichung Veterans General Hospital (CE13151-1).

### Study population

**Fig 1:**
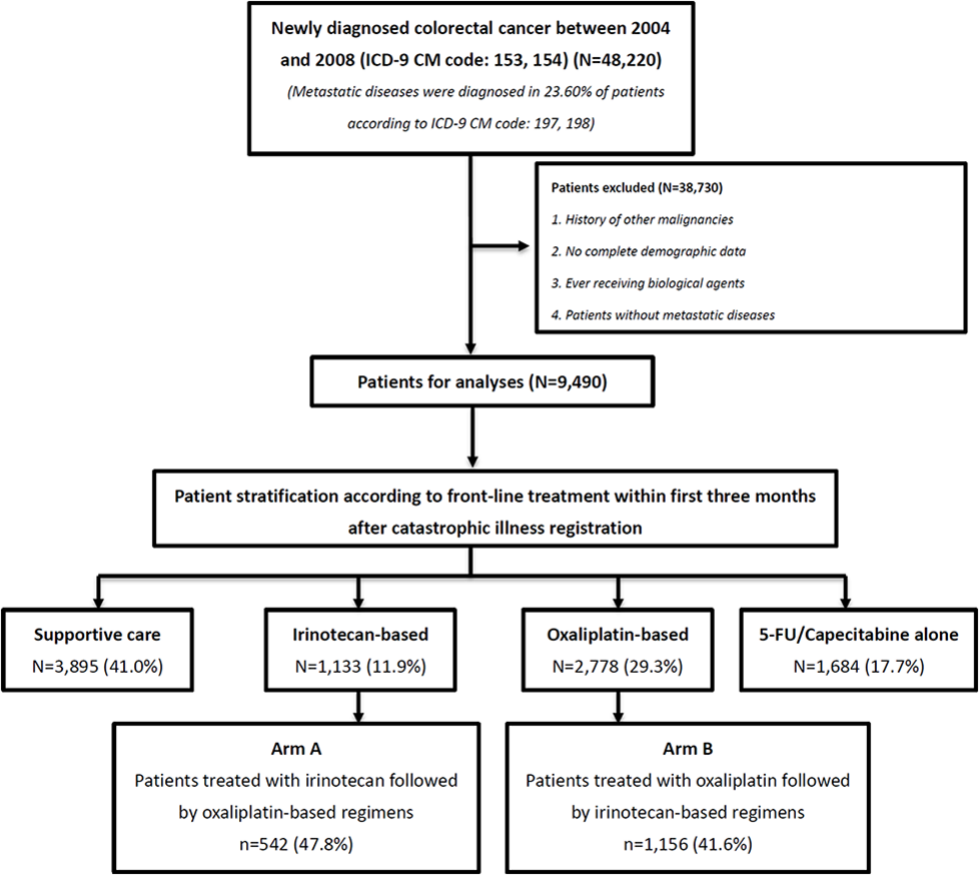
Patient selection and stratification.

Patient selection criteria are shown in Fig 1. Patients with colorectal cancer newly diagnosed between 2004 and 2008 (n = 48220) were identified using ICD-9-CM codes 153 and 154. Colorectal cancer diagnosis was further confirmed by catastrophic illness registration. Among these 48220 colorectal cancer patients, 11356 (23.6%) patients diagnosed with stage IV disease were identified using the ICD-9-CM codes 197 and 198. To further validate the use of ICD-9-CM diagnosis codes for patient identification, the distribution of stage IV disease in our study cohort and Taiwan’s national cancer registration data were compared and found to be similar [9]. Patients who had received biological agents and patients with non-metastatic disease, other malignancies, or incomplete demographic data were excluded (n = 38730). Thus, a total of 9490 mCRC patients were included in the analysis. To eliminate lead-time bias as much as possible, patients who did not undergo chemotherapy within the first three months after catastrophic illness registration were considered to have received best supportive care only (3895/9490, 41.04%). Patients who underwent chemotherapy within the first three months after catastrophic illness registration were stratified into three groups according to front-line therapies, which were irinotecan-based regimens (1133/9490, 11.94%), oxaliplatin-based regimens, (2778/9490, 29.27%), and 5-fluorouracil/capecitabine alone (1684/9490, 17.74%). Patients who received irinotecan followed by oxaliplatin-based regimens and those who received the reverse sequence were further stratified into arm A (n = 542) and B (n = 1156), respectively. The median follow-up time for patients in arm A and arm B was 594 days and 550 days, respectively (p = 0 .07).

### Comorbidities and outcome measures

Hypertension (ICD-9-CM codes 401–405), diabetes (ICD-9-CM code 250), hyperlipidemia (ICD-9-CM code 272), cardiovascular disease (ICD-9-CM codes 390–438), and chronic kidney disease (ICD-9-CM code 585) were considered major comorbidities. Patients diagnosed with primary or secondary comorbidities within one year of the date of catastrophic illness registration (outpatient or inpatient care) were considered to have comorbidities. Charlson comorbidity index (CCI) was also used to evaluate overall comorbidity.[10]

Outcome measures included first time to next treatment (TTNT1), second time to next treatment (TTNT2), and OS. TTNT1 was defined as the period between the initiation of first and second-line chemotherapy. TTNT2 was defined as the period between initiation and cessation of second-line chemotherapy. OS time was defined as the time from the initiation of first-line chemotherapy to the time of death.

### Statistical analysis

The Chi-square test, Student’s t-test, and analysis of variance were used to compare clinical variables among groups. To examine the relationship between treatment sequence and survival, multivariate Cox proportional hazards models, stratified by age, gender, and comorbidities, were used to calculate adjusted hazard ratios (HRs) and their 95% confidence intervals (CIs). TTNT1, TTNT2, and OS were compared between arm A and arm B using the Wilcoxon rank sum test. Two-tailed p values < 0.05 were considered statistically significant. All statistical analyses were performed using SAS statistical software (version 9.2 for Windows; SAS Institute Inc., Cary, NC, USA).

## Results

### Real-world treatment of mCRC in Taiwan

Of 9490 mCRC patients, 5595 (58.96%) patients were treated with chemotherapy within the first three months after catastrophic illness registration. Among these 5595 patients, 1133 (20.25%) and 2778 (49.65%) received front-line irinotecan and oxaliplatin-based chemotherapy, respectively. Notably, 5-fluorouracil or capecitabine alone was the front-line treatment in 1684 (30.10%) patients.

Of 9490 mCRC patients, 3895 (41.04%) did not receive any chemotherapeutic agents within the first three months after catastrophic illness registration. Therefore, clinical characteristics were compared between patients receiving and not receiving palliative chemotherapy (Table 1). Patients who were treated with best supportive care were older (p< 0.001) and had higher CCIs (p<0.001) and incidences of hypertension (p<0.001), diabetes (p<0.001), cardiovascular disease (p<0.001), and chronic kidney disease (p<0.001) than those who were treated with irinotecan-based regimens, oxaliplatin-based regimens, and 5-fluorouracil/capecitabine alone, indicating that comorbidities could be a major deterrent for mCRC patients to undergo chemotherapy. In addition, age, gender, and comorbidities were very similar between patients who received front-line irinotecan and oxaliplatin-based regimens, suggesting that these clinical characteristics are not useful indicators for the selection of front-line irinotecan or oxaliplatin-based regimens in mCRC patients.

**Table 1:** Comparison of Clinical Characteristics of Metastatic Colorectal Cancer Patients According to Front-line Treatment.

	Total			Supportive care		5-Fluorouracil or Capecitabine alone		Irinotecan-based		Oxaliplatin-based		p value
	(n = 9490)			(n = 3895)		(n = 1684)		(n = 1133)		(n = 2778)		
	n (%)			n (%)		n (%)		n (%)		n (%)		
**Age, years (mean ± SD)**	65.4 ± 14.2			71.5 ± 12.9		64.2 ± 13.7		59.2 ± 12.8		60.2 ± 13.5		<0.001^a^
<40	465	(4.9)		82	(2.1)	81	(4.8)	82	(7.2)	220	(7.9)	<0.001^b^
40−59	2683	(28.3)		618	(15.9)	515	(30.6)	468	(41.3)	1082	(38.9)	
≧60	6342	(66.8)		3195	(82.0)	1088	(64.6)	583	(51.5)	1476	(53.1)	
**Gender**												0.39^b^
Female	4101		(43.2)	1714	(44.0)	730	(43.3)	467	(41.2)	1190	(42.8)	
Male	5389		(56.8)	2181	(56.0)	954	(56.7)	666	(58.8)	1588	(57.2)	
**CCI (mean ± SD)**	1.1 ± 1.7			1.4 ± 1.9		1.0 ± 1.7		0.8 ± 1.4		0.8 ± 1.5		<0.001^a^
0	4914		(51.8)	1687	(43.3)	896	(53.2)	666	(58.8)	1665	(59.9)	<0.001^b^
1−2	3267		(34.4)	1497	(38.4)	573	(34.0)	359	(31.7)	838	(30.2)	
≧3	1309		(13.8)	711	(18.3)	215	(12.8)	108	(9.5)	275	(9.9)	
**Hypertension**												<0.001^b^
No	5961		(62.8)	2141	(55.0)	1105	(65.6)	803	(70.9)	1912	(68.8)	
Yes	3529		(37.2)	1754	(45.0)	579	(34.4)	330	(29.1)	866	(31.2)	
**Diabetes**												<0.001^b^
No	7751		(81.7)	3053	(78.4)	1372	(81.5)	971	(85.7)	2355	(84.8)	
Yes	1739		(18.3)	842	(21.6)	312	(18.5)	162	(14.3)	423	(15.2)	
**Hyperlipidemia**												0.12^b^
No	8292		(87.4)	3425	(87.9)	1463	(86.9)	1005	(88.7)	2399	(86.4)	
Yes	1198		(12.6)	470	(12.1)	221	(13.1)	128	(11.3)	379	(13.6)	
**CVD**												<0.001^b^
No	5260		(55.4)	1813	(46.5)	983	(58.4)	728	(64.3)	1736	(62.5)	
Yes	4230		(44.6)	2082	(53.5)	701	(41.6)	405	(35.7)	1042	(37.5)	
**CKD**												<0.001^b^
No	9289		(97.9)	3763	(96.6)	1661	(98.6)	1121	(98.9)	2744	(98.8)	
Yes	201		(2.1)	132	(3.4)	23	(1.4)	12	(1.1)	34	(1.2)	

SD: standard deviation; CCI: Charlson comorbidity index; CVD: cardiovascular disease; CKD: chronic kidney disease.

^a^ANOVA for continuous variables

^b^Chi-square test for categorical variables.

### Patients treated with irinotecan followed by oxaliplatin-based regimens had a higher crossover rate than those treated with the reverse sequence

To determine the appropriate sequence of irinotecan and oxaliplatin-based regimens for mCRC, we compared the crossover rate between mCRC patients who received irinotecan followed by oxaliplatin-based regimens (arm A; n = 542) and those who received the reverse sequence (arm B; n = 1156). Crossover was defined as delivery of the other cytotoxic agent at least twice within two months. Clinical characteristics including age (p = 0.67), gender (p = 0.95), CCI (p = 0.78), and incidences of hypertension (p = 0.68), diabetes (p = 0.95), hyperlipidemia (p = 0.22), cardiovascular disease (p = 0.90), and chronic kidney disease (p = 0.46) were not significantly different between the two arms (Table 2). Although less than half of the patients in both arms could cross over to the second-line treatment, the crossover rate was higher in arm A than in arm B (47.84% [542/1133] vs. 41.61% [1156/2778]; p<0.001).

**Table 2:** Comparison of Clinical Characteristics of Patients in Arm A and Arm B^a^.

	Total (n = 1698)		Arm A (n = 542)		Arm B (n = 1156)		p value
	n (%)		n (%)		n (%)		
**Age, years (mean ± SD)**	58.2 ± 12.8		58.4 ± 12.3		58.1 ± 13.0		0.67^b^
<40	133	(7.8)	34	(6.3)	99	(8.6)	0.23^c^
40−59	755	(44.5)	240	(44.3)	515	(44.6)	
≥60	810	(47.7)	268	(49.4)	542	(46.9)	
**Gender**							0.95^c^
Female	738	(43.5)	235	(43.4)	503	(43.5)	
Male	960	(56.5)	307	(56.6)	653	(56.5)	
**CCI (mean ± SD)**	0.8 ± 1.3		0.7 ± 1.1		0.8 ± 1.4		0.78^b^
0	1026	(60.4)	315	(58.1)	711	(61.5)	0.18^c^
1−2	513	(30.2)	180	(33.2)	333	(28.8)	
≥3	159	(9.4)	47	(8.7)	112	(9.7)	
**Hypertension**							0.68^c^
No	1182	(69.6)	381	(70.3)	801	(69.3)	
Yes	516	(30.4)	161	(29.7)	355	(30.7)	
**Diabetes**							0.95^c^
No	1455	(85.7)	464	(85.6)	991	(85.7)	
Yes	243	(14.3)	78	(14.4)	165	(14.3)	
**Hyperlipidemia**							0.22^c^
No	1469	(86.5)	477	(88.0)	992	(85.8)	
Yes	229	(13.5)	65	(12.0)	164	(14.2)	
**CVD**							0.90^c^
No	1077	(63.4)	345	(63.7)	732	(63.3)	
Yes	621	(36.6)	197	(36.3)	424	(36.7)	
**CKD**							0.46^c^
No	1681	(99.0)	538	(99.3)	1143	(98.9)	
Yes	17	(1.0)	4	(0.7)	13	(1.1)	

^a^Arm A, irinotecan followed by oxaliplatin-based regimens; arm B, oxaliplatin followed by irinotecan-based regimens.

SD: standard deviation; CCI: Charlson comorbidity index; CVD: cardiovascular disease; CKD: chronic kidney disease.

^b^Student’s t-test for continuous variables

^c^Chi-square test for categorical variables.

### TTNT2 and OS, but not TTNT1, were better in patients receiving irinotecan followed by oxaliplatin-based regimens than in patients receiving the reverse sequence

**Fig 2:**
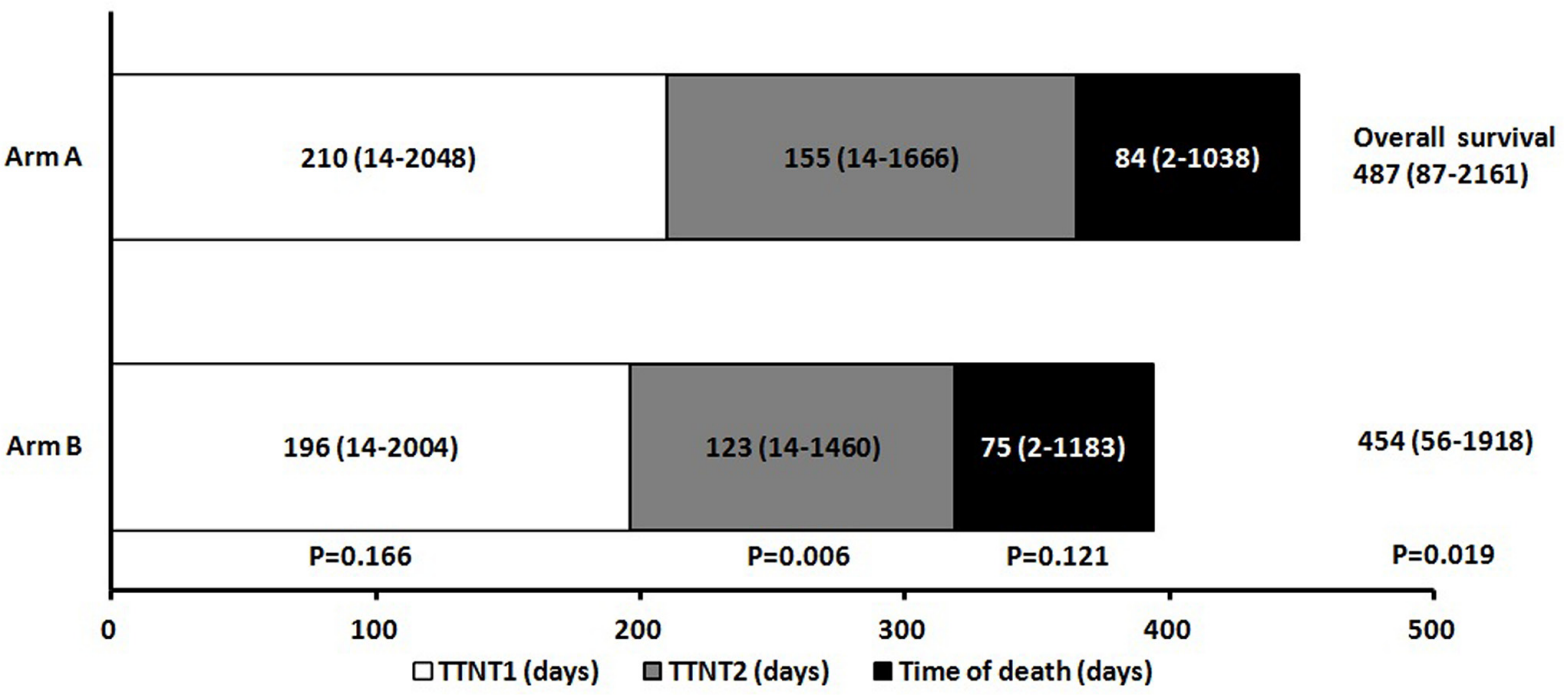
Comparison of survival in metastatic colorectal cancer patients treated with irinotecan followed by oxaliplatin-based regimens or the reverse sequence. The median first time to next treatment (TTNT1) in arm A (irinotecan followed by oxaliplatin-based regimens) was 210 days (14−2048). It was 196 days (14−2004) in arm B (oxaliplatin followed by irinotecan-based regimens). TTNT1 was not significantly different between patients in arm A and arm B (p = 0.17). Moreover, the median second time to next treatment (TTNT2) in arm A and arm B was 155 days (14−1666) and 123 days (14−1460), respectively. TTNT2 was longer for patients in arm A than for those in arm B (p = 0.006). In terms of overall survival (OS), the median OS time for arm A and arm B was 487 days (87−2161) and 454 days (56−1918), respectively. OS was significantly longer for patients in arm A than for those in arm B (p = 0.02).

A comparison of TTNT1, TTNT2, and OS between arms A and B is shown in Fig 2. The median TTNT1 was similar between the two arms (p = 0.17) and was 210 days (range: 14−2048 days) in arm A and 196 days (range: 14−2004 days) in arm B. The median TTNT2 was significantly longer in arm A than in arm B (155 days vs. 123 days; p = 0.006). Time from cessation of chemotherapy to death was not significantly different between arm A and arm B (84 days vs. 75 days; p = 0.12). Notably, patients in arm A had a superior OS than those in arm B (p = 0.02). The median OS time for patients in arm A and arm B was 487 days (range: 87−2161 days) and 454 days (range: 56−1918), respectively.

### Irinotecan and oxaliplatin-based chemotherapy sequences yielded similar OS in mCRC patients

**Fig 3:**
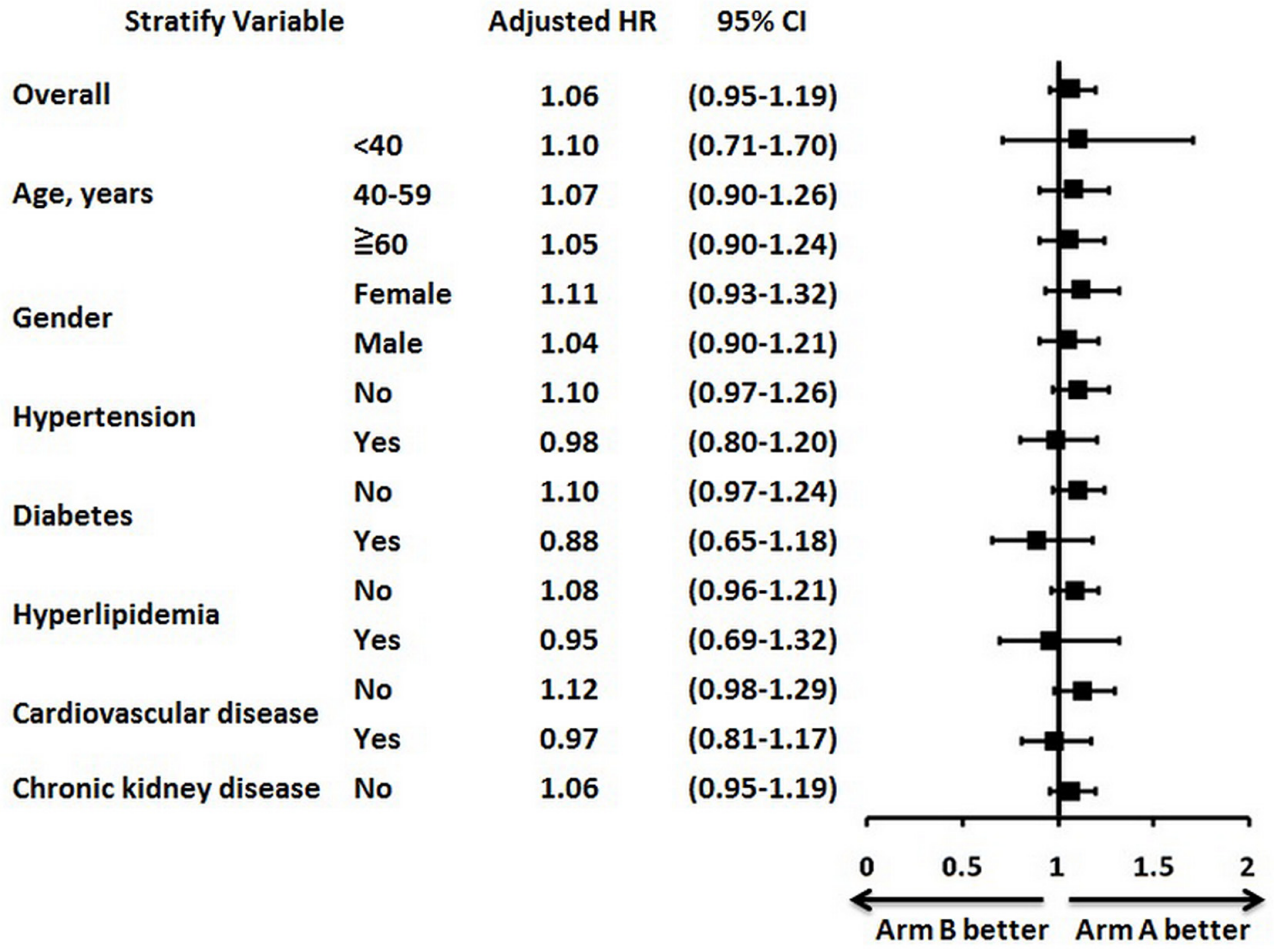
Subgroup analyses of overall survival for oxaliplatin followed by irinotecan-based regimens versus the reverse sequence. The overall hazard ratio (HR) for oxaliplatin followed by irinotecan-based regimens (arm A) versus the reverse sequence (arm B) was 1.06 (95% confidence interval [CI]: 0.95−1.19; p = 0.27). Age, gender, hypertension, diabetes, hyperlipidemia, cardiovascular disease, and chronic kidney disease were not independently associated with better overall survival in patients receiving either chemotherapy sequence.

Cox proportional hazards regression adjusted by age, gender, hypertension, diabetes, hyperlipidemia, cardiovascular disease, and chronic kidney disease was used to identify patients who may derive greater benefit from front-line irinotecan-based regimens followed by second-line oxaliplatin-based regimens or the reverse sequence (Fig 3). The overall HR for oxaliplatin followed by irinotecan-based regimens versus the reverse sequence was 1.06 (95% CI: 0.95−1.19; p = 0.27), suggesting that OS was comparable between these two treatment sequences. Furthermore, age, gender, and comorbidities (hypertension, diabetes, hyperlipidemia, cardiovascular disease, and chronic kidney disease) were not independently associated with better OS in patients treated with either irinotecan followed by oxaliplatin-based regimens or the reverse sequence.

## Discussion

Although palliative chemotherapy is a standard of care for mCRC, our nationwide population-based study showed that less of 60% of mCRC patients in Taiwan received chemotherapeutic treatment within the first three months after diagnosis. Comorbidities such as hypertension, diabetes, cardiovascular disease, and chronic kidney disease were the major reason for mCRC patients not to undergo palliative chemotherapy. Promising OS benefit not necessarily obtained by early chemotherapy could be another possibility because a meta-analysis by Ackland et al. [11] showed that OS is not significantly different between asymptomatic mCRC patients who received immediate or delayed chemotherapy, suggesting that immediate chemotherapy might not always be needed for mCRC patients. Additionally, although multiple phase III trials have shown that doublet chemotherapy with 5-fluorouracil and irinotecan/oxaliplatin provides both superior PFS and OS compared with monotherapy with 5-fluorouracil in mCRC patients [3, 12], 5-fluorouracil or capecitabine alone remained one of the front-line treatment options in our study cohort. These data suggest that the adverse effects of doublet chemotherapy regimens are still of concern to physicians and patients in a real-world practice.

We aimed to determine the best sequence of irinotecan and oxaliplatin-based regimens for mCRC. Our study results showed that TTNT1 was comparable between the two treatment sequences. Our finding was at least partially supported with a previous phase III randomized trial showing that the same median time to progression (7 months) was achieved with FOLFIRI and FOLFOX4 regimens in mCRC patients [13]. In terms of second-line chemotherapy, our study results demonstrated that TTNT2 was significantly longer in patients in arm A than in patients in arm B (155 days vs. 123 days). In a GERCOR study [5], median PFS was longer in patients who received second-line FOLFOX6 than in patients who received second-line FOLFIRI (4.2 months vs. 2.5 months). In addition, chemotherapy-associated side effects were comparable between the two groups, except for the higher neuropathy rate in the FOLFOX6 group. Importantly, the longer TNTT2 may have contributed to the better overall survival in mCRC patients receiving irinotecan followed by oxaliplatin-based regimens in our study. Our findings suggest that irinotecan-based regimens should be used as first-line chemotherapy instead of oxaliplatin-based regimens in patients with mCRC.

Although mCRC patients receiving front-line irinotecan-based regimens had a better OS compared with those receiving front-line oxaliplatin-based regimens, the multivariate Cox proportional hazards regression analysis failed to confirm the superior survival rate of front-line irinotecan-based regimens. The overall HR for oxaliplatin followed by irinotecan-based regimens versus the reverse sequence was not significant, suggesting that the two sequences provide a similar survival benefit in mCRC patients. Oxaliplatin-based chemotherapy has been shown to be more beneficial in *KRAS*-mutated mCRC than in *KRAS* wild-type mCRC [14]. In our study, we were unable to identify any clinical variables that might be useful in selecting the appropriate chemotherapy sequencing approach. Age, gender, hypertension, diabetes, hyperlipidemia, cardiovascular disease, and chronic kidney disease were not associated with OS in either treatment arm. Notably, the crossover rate was higher for patients treated with front-line irinotecan-based regimens than for those treated with front-line oxaliplatin-based regimens. The higher rate of neuropathy in patients receiving front-line oxaliplatin may explain the higher crossover rate for front-line irinotecan-based regimens.

The major limitation of the current study was the retrospective study design. In addition, our study did not investigate the impact of biological therapies on outcome. Whether the addition of biological agents to sequential cytotoxic regimens would have influenced our study results is unclear. A future subgroup analysis from the United States intergroup phase III C80405 trial of combined cetuximab/bevacizumab and FOLFOX/FOLFIRI will help address this issue [15].

## Conclusions

Our study showed that less than 60% of mCRC patients in Taiwan received early palliative chemotherapy. Older patients and those with higher CCIs, hypertension, diabetes, cardiovascular disease, and chronic kidney disease preferred to receive best supportive care. The crossover rate was higher for patients treated with front-line irinotecan-based regimens followed by second-line oxaliplatin-based regimens than for those treated with the reverse sequence. The higher crossover rate and longer TTNT2 for irinotecan followed by oxaliplatin-based regimens may translate into an OS benefit in mCRC patients. Our study not only presented a real-world treatment of mCRC before the era of biological agents, but also provided a reasonable strategy for choosing the optimal chemotherapeutic backbone for the integration of newly developed biological agents. Data from studies with prospective and randomized-controlled designs, however, are required for more solid conclusions.

## References

[1] Jemal A, Siegel R, Xu J, Ward E. Cancer statistics, 2010. CA: a cancer journal for clinicians. 2010;60(5):277–300. Epub 2010/07/09. 10.3322/caac.20073 . 20610543

[2] Goldberg RM, Gill S. Recent phase III trials of fluorouracil, irinotecan, and oxaliplatin as chemotherapy for metastatic colorectal cancer. Cancer chemotherapy and pharmacology. 2004;54 Suppl 1:S57–64. Epub 2004/08/17. 10.1007/s00280-004-0888-9 . 15309516

[3] de Gramont A, Figer A, Seymour M, Homerin M, Hmissi A, Cassidy J, et al Leucovorin and fluorouracil with or without oxaliplatin as first-line treatment in advanced colorectal cancer. Journal of clinical oncology: official journal of the American Society of Clinical Oncology. 2000;18(16):2938–47. Epub 2000/08/16. . 10944126 10.1200/JCO.2000.18.16.2938

[4] Saltz LB, Cox JV, Blanke C, Rosen LS, Fehrenbacher L, Moore MJ, et al Irinotecan plus fluorouracil and leucovorin for metastatic colorectal cancer. Irinotecan Study Group. The New England journal of medicine. 2000;343(13):905–14. Epub 2000/09/28. 10.1056/NEJM200009283431302 . 11006366

[5] Tournigand C, Andre T, Achille E, Lledo G, Flesh M, Mery-Mignard D, et al FOLFIRI followed by FOLFOX6 or the reverse sequence in advanced colorectal cancer: a randomized GERCOR study. Journal of clinical oncology: official journal of the American Society of Clinical Oncology. 2004;22(2):229–37. Epub 2003/12/06. 10.1200/JCO.2004.05.113 . 14657227

[6] Hurwitz H, Fehrenbacher L, Novotny W, Cartwright T, Hainsworth J, Heim W, et al Bevacizumab plus irinotecan, fluorouracil, and leucovorin for metastatic colorectal cancer. The New England journal of medicine. 2004;350(23):2335–42. Epub 2004/06/04. 10.1056/NEJMoa032691 . 15175435

[7] Van Cutsem E, Kohne CH, Hitre E, Zaluski J, Chang Chien CR, Makhson A, et al Cetuximab and chemotherapy as initial treatment for metastatic colorectal cancer. The New England journal of medicine. 2009;360(14):1408–17. Epub 2009/04/03. 10.1056/NEJMoa0805019 . 19339720

[8] Lin CH, Sheu WH. Hypoglycaemic episodes and risk of dementia in diabetes mellitus: 7-year follow-up study. Journal of internal medicine. 2013;273(1):102–10. Epub 2012/09/26. 10.1111/joim.12000 . 23003116

[9] Teng CL, Yu JT, Chen YH, Lin CH, Hwang WL. Early colonoscopy confers survival benefits on colon cancer patients with pre-existing iron deficiency anemia: a nationwide population-based study. PloS one. 2014;9(1):e86714 Epub 2014/01/28. 10.1371/journal.pone.0086714 24466209 PMC3899285

[10] Charlson ME, Pompei P, Ales KL, MacKenzie CR. A new method of classifying prognostic comorbidity in longitudinal studies: development and validation. Journal of chronic diseases. 1987;40(5):373–83. Epub 1987/01/01. . 3558716 10.1016/0021-9681(87)90171-8

[11] Ackland SP, Jones M, Tu D, Simes J, Yuen J, Sargeant AM, et al A meta-analysis of two randomised trials of early chemotherapy in asymptomatic metastatic colorectal cancer. British journal of cancer. 2005;93(11):1236–43. 10.1038/sj.bjc.6602841 16265352 PMC2361520

[12] Douillard JY, Cunningham D, Roth AD, Navarro M, James RD, Karasek P, et al Irinotecan combined with fluorouracil compared with fluorouracil alone as first-line treatment for metastatic colorectal cancer: a multicentre randomised trial. Lancet. 2000;355(9209):1041–7. . 10744089 10.1016/s0140-6736(00)02034-1

[13] Colucci G, Gebbia V, Paoletti G, Giuliani F, Caruso M, Gebbia N, et al Phase III randomized trial of FOLFIRI versus FOLFOX4 in the treatment of advanced colorectal cancer: a multicenter study of the Gruppo Oncologico Dell'Italia Meridionale. Journal of clinical oncology: official journal of the American Society of Clinical Oncology. 2005;23(22):4866–75. 10.1200/JCO.2005.07.113 . 15939922

[14] Lin YL, Liang YH, Tsai JH, Liau JY, Liang JT, Lin BR, et al Oxaliplatin-based chemotherapy is more beneficial in KRAS mutant than in KRAS wild-type metastatic colorectal cancer patients. PloS one. 2014;9(2):e86789 10.1371/journal.pone.0086789 24505265 PMC3913571

[15] CALGB/SWOG C80405: A phase III trial of FOLFIRI or FOLFOX with bevacizumab or cetuximab or both for untreated metastatic adenocarcinoma of the colon or rectum. Clinical advances in hematology & oncology: H&O. 2006;4(6):452–3. . 16981668

